# Catheter-related complications in AML remission induction chemotherapy: a retrospective comparison of PICCs and CICCs

**DOI:** 10.1007/s00520-026-11146-3

**Published:** 2026-08-28

**Authors:** S. Ehrlich, F. Aytan, J. Eufinger, M. Ruzicka, D. Doedens, M. Seidensticker, T. Herold, M. Subklewe, V. Bücklein, M. von Bergwelt-Baildon, K. Spiekermann

**Affiliations:** 1 Department of Medicine III, University Hospital, Munich, Germany; 2 Department of Oral and Maxillofacial Surgery, TUM University Hospital Klinikum Rechts der Isar, School of Medicine and Health, Technical University of Munich, Munich, Germany; 3 Department of Radiology, University Hospital, Munich, Germany; 4 Department of Hematology, Oncology, and Stem-Cell Transplantation, Munich Hospital Bogenhausen, Munich Municipal Hospital Group, Munich, Germany; 5 German Cancer Consortium (DKTK), Partner Site Munich, Munich, Germany; 6 Bavarian Cancer Research Center (BZKF), Munich, Germany; 7 Comprehensive Cancer Center Munich (CCC), Munich, Germany

**Keywords:** Central venous catheter, PICC, CICC, AML, CRBSI

## Abstract

**Purpose:**

Central venous catheters (CVCs) are a cornerstone for effective supportive therapy in patients with AML undergoing remission induction chemotherapy. While peripherally inserted central catheters (PICCs) are increasingly used as an alternative to centrally inserted central catheters (CICCs), real world data on their implications on AML and supportive therapy remain limited. This study aimed to compare infectious and non-infectious complications of PICCs vs. CICCs in this population.

**Methods:**

We conducted a single-center retrospective cohort study of 116 AML patients receiving induction chemotherapy between June 2019 and March 2024. Patients received either a PICC (*n* = 59) or a CICC (*n* = 57). Catheter-related complications were analyzed as incidence rates per 1000 catheter days.

**Results:**

PICCs had a longer median dwell time than CICCs (16 vs. 11 days, *p* = 0.004). Catheter-related bloodstream infection (CRBSI) rates were low and similar (2.8 vs. 2.6/1000 catheter days, *p* = 0.93). Exit site infections were significantly lower in the PICC group (7.4 vs. 23.1/1000 catheter days, *p* = 0.01). Bleeding events occurred more frequently in PICCs (13.9 vs. 6.4/1000 catheter days, *p* = 0.14) but were mostly minor. More patients in the CICC group received at least one platelet transfusion before insertion (36.8% vs. 16.9%, *p* = 0.03). Mechanical complications and symptomatic thromboses were rare in both groups.

**Conclusion:**

PICCs were associated with longer dwell times and fewer local infections, without increased risk of CRBSIs or thrombosis. These findings suggest that PICCs may help optimize supportive care in clinically stable patients undergoing AML induction chemotherapy.

## Introduction

In recent years, the prognosis of patients with acute myeloid leukemia (AML) has improved substantially owing to the introduction of novel intensive treatment strategies, targeted molecular therapies, and, importantly, major advances in supportive care [1]. Nevertheless, patients undergoing intensive remission induction chemotherapy continue to experience prolonged and profound neutropenia, during which severe infections remain common and are associated with considerable morbidity and mortality [2].

For this reason, effective supportive care is indispensable. In addition to anti-infective prophylaxis, prompt diagnostic evaluation and immediate initiation of empirical antibiotic therapy for febrile neutropenia, reliable central venous access is essential. Central venous catheters (CVCs) are required not only for the administration of chemotherapy but also facilitate daily blood sampling, blood product transfusions, prolonged intravenous antimicrobial therapy, and, when necessary, parenteral nutrition [3]. Consequently, catheter-associated complications may lead to relevant interruptions of supportive care, additional invasive procedures for catheter replacement, prolonged hospitalization, and delays in leukemia treatment.

Besides conventional centrally inserted central catheters (CICCs), which are mostly inserted in the internal jugular or subclavian vein, peripherally inserted central catheters (PICCs), have become increasingly established over recent years. PICCs represent an attractive alternative because they are inserted into the basilic or brachial vein of the upper arm, thereby avoiding puncture of the neck or thorax. Furthermore, patients can be discharged with the catheter in place and continue treatment in the outpatient setting [4]. For these reasons, PICCs are often preferred by patients. Evidence comparing PICCs and CICCs during AML remission induction chemotherapy remains limited and inconsistent. While some studies reported lower rates of catheter-related infections and comparable or even reduced thrombotic complications with PICCs [5, 6], others found a significantly increased risk of catheter-related thrombosis [7].

However, there is still limited real-world evidence on the effects of these two catheter types on supportive care during intensive remission induction chemotherapy. In addition to catheter-related bloodstream infections and thrombosis, clinically relevant outcomes include catheter dwell time, local catheter-related infections, bleeding complications, and the need for transfusions associated with catheter insertion.

Therefore, this study aimed to compare PICCs and CICCs in a contemporary real-world cohort of adults with AML undergoing remission induction chemotherapy, with particular emphasis on clinically relevant catheter-related complications and their implications for supportive care.

## Patients and methods

### Study population and design

We conducted a single-center retrospective cohort study at LMU University Hospital, Munich, a tertiary care center in Germany. All adult patients (≥ 18 years) with newly diagnosed AML who received intensive remission induction chemotherapy between June 2019 and March 2024 and either a CICC or PICC were eligible. Patients with acute promyelocytic leukemia, additional CVC (e.g. port-catheter) or central catheters other than PICC or CICC (e.g. Sheldon-catheter) were excluded. Only the first central catheter was considered for analysis. The observation period began at the day of catheter placement and ended on the day of catheter removal.

This study was performed in line with the principles of the 1964 Declaration of Helsinki and its later amendments. Approval was granted by the Ethics Committee of the LMU University (23–0091).

### Catheter placement

All patients undergoing intensive remission induction chemotherapy routinely received a central venous access device for the administration of cytotoxic therapy. The type of catheter (PICC vs. CICC) was chosen based on patient preference, feasibility, and the decision of the treating physician. All central venous catheters (CVCs) were placed according to standardized institutional protocols for safe and sterile insertion. Surgical hand disinfection, sterile gloves, long-sleeved surgical gowns, caps, and face masks were used as part of maximal sterile barrier precautions. Skin antisepsis before catheter insertion was performed using either an alcohol- or an iodine-based antiseptic and then covered with sterile drapes. Chlorhexidine-impregnated dressings were used routinely. Catheterization was performed under real-time ultrasound guidance using the Seldinger technique. For fixation, sutures were used for CICCs, whereas a sutureless securement device was used for PICCs. The catheter tip position was confirmed at the cavoatrial junction or in the lower third of the superior vena cava using electrocardiography and chest X-ray. PICCs were routinely placed in the radiology department, while CICCs were inserted by experienced anesthesiologists or experienced physicians of the intensive care unit or the hematology ward. All patients received antifungal prophylaxis, but adhering to hospital guidelines, antibacterial prophylaxis was omitted. Patients additionally received acyclovir 400 mg twice daily and in exceptional cases also cotrimoxazole 960 mg twice daily two days per week as PCP prophylaxis. In case of platelet counts > 30 × 10^9^/L prophylactic anticoagulation with low molecular weight heparin was administered.

### Data collection, definitions, and infection assessment

The electronic medical records of patients were reviewed to collect data on demographics, chemotherapy regimens, radiological, and microbiological findings. Patients were monitored closely for signs of local or systemic infections at the exit site or thrombosis.

Fever was defined as a single temperature of ≥ 38.3 °C or a temperature of ≥ 38.0 °C lasting for more than 1 h. Neutropenia was defined as an absolute neutrophil count (ANC) < 500/µl, < 1000/µl with a predicted decline to < 500/µl in the next 48 h, or a white blood cell count (WBC) < 1000/µl if a differential was not available. In cases of fever, diagnostic workup was conducted in accordance with the national guidelines [8]. This included at least two sets of blood cultures and additional diagnostic measures depending on the suspected site of infection. Central catheters were removed in case of signs of exit site infection, in neutropenic fever with no other focus, if no longer needed, or if dysfunctional. Whenever a catheter was removed and an infection was suspected, its tip was submitted for microbiological examination. Empirical antibacterial therapy was initiated immediately, and anti-infective treatment was subsequently adjusted if a pathogen was identified.

Exit site infection was defined as clinical signs of inflammation at the catheter insertion site, including erythema, warmth, swelling, pain, or purulent discharge, with or without evidence of bloodstream infection or microbiological confirmation.

Bloodstream infection was defined as isolation of a pathogenic microorganism from at least one blood culture accompanied by clinical signs of infection, irrespective of the presumed infectious focus. In contrast, isolation of typical skin bacteria in only a single blood culture was considered contamination and was not counted as a bloodstream infection.

CVCs were assessed for CRBSIs based on the 2012 criteria of the Infectious Diseases Working Party (AGIHO) of the German Society of Hematology and Medical Oncology (DGHO) [9]. Both definite and probable CRBSIs were included in the analysis. Definite CRBSI was diagnosed in cases of either (i) identical pathogens cultured from the catheter tip and peripheral blood, or (ii) identical organisms detected in blood cultures from the central line and a peripheral vein, with a differential time to positivity (DTTP) > 2 h or at least a threefold higher colony count in central vs. peripheral cultures. Probable CRBSI was defined as the presence of typical skin or catheter-associated pathogens (e.g., coagulase-negative staphylococci, Staphylococcus aureus, or Candida spp.) in both central and peripheral blood cultures, in the absence of an alternative infectious source, not fulfilling the criteria for definite CRBSI.

Catheter-associated thrombosis was considered present if a thrombus was documented in the medical records by imaging (Doppler ultrasound, computed tomography [CT]) in a vein where a central venous catheter was placed, irrespective of symptoms. Symptomatic catheter-associated thrombosis was defined as the radiologically confirmed presence of a thrombus in a vein used for central venous catheter placement, in combination with clinical symptoms such as swelling, pain, erythema, or functional impairment of the affected limb or region.

### Outcomes

The primary outcomes were the catheter dwell time and the rate of CRBSIs per 1000 catheter days. Other secondary endpoints included local infections, bleeding after CVC insertion, catheter-related venous thrombosis, and catheter dysfunction during follow-up.

### Statistical analysis

Baseline characteristics of patients receiving a peripherally inserted central catheter (PICC) versus a centrally inserted central catheter (CICC) were compared using descriptive statistics. Categorical variables were presented as counts and percentages and compared using Fisher’s exact or χ^2^ tests, as appropriate. Continuous variables were reported as medians with interquartile ranges (IQRs) and compared using the Wilcoxon rank-sum test.

Catheter-related outcomes, including CRBSIs and other complications, were expressed as incidence rates per 1000 catheter days. To evaluate the association between clinical variables and the incidence of catheter-related complications, an univariable Poisson regression model was applied. Results were reported as incidence rate ratios (IRRs) with 95% confidence intervals (CIs). 95% CIs were calculated using the Wald method based on model-based standard errors (exp[β ± 1.96 × SE]). For endpoints with zero events in one group, IRR was not estimable; Fisher’s exact test *p*-values are reported.

All statistical tests were two-sided, and a *p*-value < 0.05 was considered statistically significant. Analyses were performed using R version 4.5.0 (R Foundation for Statistical Computing, Vienna, Austria).

## Results

### Patients’ characteristics

A total of 116 patients were included in the study, with 59 receiving a peripherally inserted central catheter (PICC) and 57 receiving a centrally inserted central catheter (CICC). Baseline characteristics between the two groups are shown in Table 1 and were largely comparable. The median age was slightly lower in the PICC group, although this difference was not statistically significant. The proportion of female patients and the distribution of de novo AML were also similar between the two groups. A significant difference was observed in ECOG (Eastern Cooperative Oncology Group) performance status, with fewer patients in the CICC group having an ECOG < 2. The proportion of patients with adverse risk AML by the ELN (European LeukemiaNet) 2017 classification was higher in the PICC group (35.6% vs. 24.6%), although this difference did not reach statistical significance (*p* = 0.275). No differences were observed in the prevalence of comorbidities like obesity or diabetes mellitus between the CICC and PICC group, respectively. Treatment regimens were equally distributed between the two groups.

**Table 1:** Patient’s characteristics

	PICC *N* = 59	CICC *N* = 57	*p*
Age, Median (IQR)	54 (40–63)	58 (52–63)	0.17
Female	27 (45.8)	27 (47.4)	1.00
Diagnosis			
de novo AML	50 (84.7)	47 (82.5)	0.93
ECOG			
< 2	57 (96.6)	49 (86.0)	0.03
ELN adverse	21 (35.6)	14 (24.6)	0.28
Comorbidities			
Adipositas	25 (42.4)	33 (57.9)	0.14
Diabetes mellitus	5 (8.5)	2 (3.5)	0.46
Chemotherapy regimen			
7 + 3 w/o targeted therapy	53 (89.8)	48 (84.2)	0.37
CPX-351	5 (8.5)	5 (8.8)	1.00
Other	1 (1.7)	4 (7.0)	0.20

If not otherwise indicated, data is shown as n (% of patients).

*IQR* (Interquartile Range), *AML* (acute myeloid leukemia), *ECOG* (Eastern Cooperative Oncology Group), *ELN* (European LeukemiaNet).

### Catheter placement

Next, we analyzed the catheter insertion settings and pre-insertion clinical parameters between the patients in the PICC and CICC group, which are shown in Table 2. PICCs were exclusively inserted in the radiology department, while CICCs were placed across various clinical settings, including the intensive or intermediate care units, anesthesiology and hematology ward. The internal jugular vein was used in all but one patient for CICCs. Most catheters were inserted on the right side in both groups.

**Table 2:** Baseline characteristics on the day of catheter insertion

	PICC *N* = 59	CICC *N* = 57	*p*
Device insertion place			
ICU/IMC	0	21 (36.8)	
Radiology department	59 (100)	0	
Hematology ward	0	13 (22.8)	
Anesthesiology	0	20 (35.1)	
na	0	3 (5.3)	
Anatomic site			
Basilic vein	46 (78.0)	0	
Brachial vein	13 (22.0)	0	
Internal jugular vein	0	56 (98.2)	
Subclavian vein	0	1 (1.8)	
Right side	52 (88.1)	51 (89.5)	1.00
Left side	7 (11.9)	6 (10.5)	
Fever prior to insertion	13 (22.0)	18 (31.6)	0.28
Interval from insertion to chemotherapy start in days, Median [IQR]	3.00 [1.00–6.00]	1.00 [0.00–4.00]	0.08
Laboratory results at baseline			
CRP in mg/L, Median [IQR]	1.80 [0.30–5.25]	3.70 [1.30–9.60]	0.03
Neutrophils in 1 × 10^9^/µL, Median [IQR]	1.00 [0.00–1.00]	0.50 [0.00–1.00]	0.17
Platelets in 1 × 10^9^/µL, Median [IQR]	36.00 [21.0–86.00]	38.00 [25.00–80.00]	0.60
Number of patients with platelet transfusion on day of insertion			
1	10 (16.9)	21 (36.8)	0.03
2	0	5 (8.8)	0.06

If not otherwise indicated, data is shown as n (% of patients).

*IQR* (Interquartile Range), *ICU (*intensive care unit), *IMC* (intermediate care), *CRP* (C-reactive protein), *na* (not available).

Fever prior to catheter insertion was documented in 13 patients (22.0%) in the PICC group and 18 patients (31.6%) in the CICC group, with no statistically significant difference. The median interval from catheter insertion to start of remission induction chemotherapy was three days in the PICC and one day in the CICC group.

CRP levels on the day of catheter insertion were higher in the CICC group compared to the PICC group, whereas neutrophil and platelet counts were similar between the groups. The number of platelet transfusions on the day of insertion was significantly higher in the CICC group, with five patients receiving more than one platelet transfusion before insertion.

### Outcomes

Catheter-related outcomes, including dwell time and infection rates, were assessed for both groups (Table 3). PICCs had a significantly longer dwell time compared to CICCs (median 16 days vs. 11 days, *p* = 0.004). CRBSI occurred at a rate of 2.8 per 1000 catheter days in the PICC group and 2.6 per 1000 catheter days in the CICC group, with an incidence rate ratio (IRR) of 1.08 with no significant difference between the groups (*p* = 0.93). CRBSIs were exclusively caused by Gram-positive bacteria in our cohort. In the PICC group, *S. haemolyticus* was identified in two patients and *S. aureus* in one patient, whereas CRBSIs in the CICC group were both caused by *S. epidermidis*.

**Table 3:** Primary and secondary endpoints

	PICC *N* = 59	CICC *N* = 57	IRR (PICC vs CICC)	95% CI	*p*
Catheter dwell time in days, Median [IQR]	16 [11.5–21.5]	11 [9–17]	-	-	0.004
Catheter days	1080	779	-	-	-
CRBSI	2.8	2.6	1.08	0.18–6.48	0.93
Bloodstream infection	17.6	14.1	1.25	0.59–2.62	0.56
Exit site infection	7.4	23.1	0.32	0.14–0.74	0.01
Bleeding	13.9	6.4	2.16	0.79–5.95	0.14
Dysfunction	2.8	3.9	0.72	0.15–3.57	0.69
Pneumothorax	0.0	0.0	-	-	1.00
Nerve injury	0.9	0.0	-	-	1.00
CRVT	1.9	0.0	-	-	0.51

If not otherwise indicated, data is shown as incidence rate per 1000 catheter days.

*IRR* (incidence rate ratio), *CI* (confidence interval), *CRBSI* (catheter-related bloodstream infection, *CRVT* (catheter-related venous thrombosis).

Next, secondary endpoints were analyzed. The incidence of overall bloodstream infections appeared slightly higher in the PICC group, even though the difference was not statistically significant. In contrast, exit site infections were significantly more prevalent in the CICC group (7.4 vs. 23.1 per 1000 catheter days, *p* = 0.01). Bleeding events were observed more often in the PICC group (13.9 vs. 6.4 per 1000 catheter days), but this difference was not statistically significant, and the bleeding was only clinically relevant and warranted further therapeutic measures in one case. If local bleeding was described, it mostly was documented directly after insertion. Catheter dysfunction occurred at low and comparable rates in both groups.

A single case of nerve injury and two cases of catheter-related venous thrombosis (CRVT) were observed in the PICC group, whereas no such events occurred in the CICC group. No pneumothorax was reported in either group.

### Risk factors for CRBSI

**Fig. 1:**
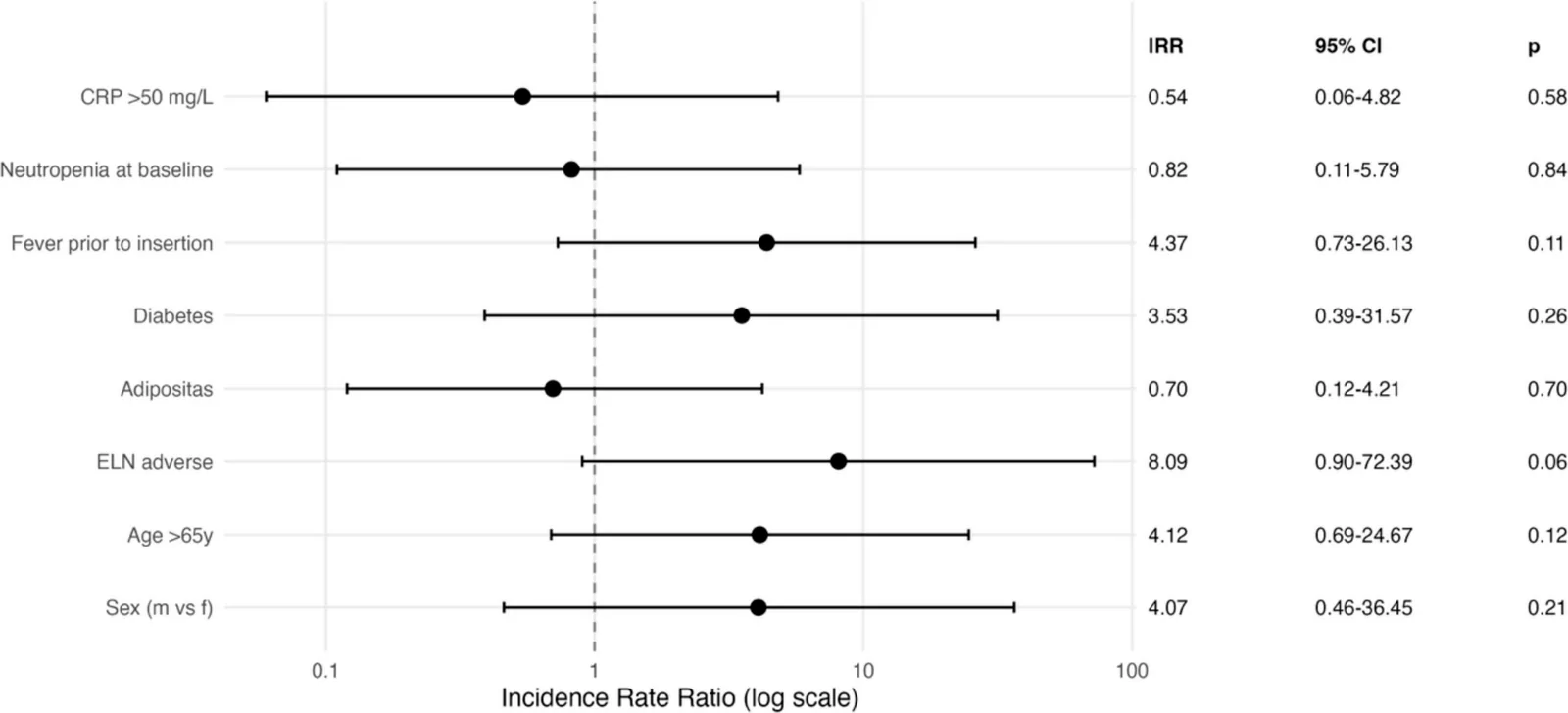
Risk Factors for CRBSIs. Univariable Poisson Regression, IRR with 95% CI. *IRR* (incidence rate ratio), *CI* (confidence interval)*, CRP* (C-reactive protein), *ELN* (European LeukemiaNet)

Due to the low number of CRBSI events (*n* = 5), multivariable analysis was not feasible. Univariate comparisons were performed for exploratory purposes only via univariable Poisson regression (Fig. 1). None of the investigated baseline characteristics showed a statistically significant association with the outcome. However, there was a trend toward higher incidence rates in patients with ELN adverse risk classification (IRR 8.09, *p* = 0.06), fever prior to insertion of catheter (IRR 4.37, *p* = 0.11), and age > 65 years (IRR 4.12, *p* = 0.12).

## Discussion

In this real-world cohort of patients with AML undergoing remission induction chemotherapy, PICCs and CICCs were associated with comparable rates of CRBSIs. However, catheter choice influenced several clinically relevant aspects of supportive care. Specifically, PICCs were associated with significantly longer catheter dwell times, fewer exit-site infections, and a lower requirement for platelet transfusions prior to catheter insertion.

Reliable central venous access is crucial for supportive care in patients undergoing intensive AML induction therapy. Besides the administration of chemotherapy, central venous catheters enable frequent blood sampling, transfusions, prolonged intravenous antimicrobial therapy, and, when required, parenteral nutrition. Consequently, catheter-related complications requiring catheter removal may influence the effectiveness of supportive care and lead to additional invasive procedures for catheter replacement, prolong hospitalization, and potentially delay leukemia treatment. Because of these reasons, the significantly longer dwell time observed with PICCs likely represents a clinically relevant advantage.

CRBSIs occurred at a low rate in both groups and did not differ significantly. This aligns with previous studies reporting comparable or even lower infection risks between catheter types in patients with hematologic malignancies, although evidence specific to AML populations remains limited [5, 6]. Catheters in our cohort were removed quite early, particularly in cases of persistent fever during neutropenia or when central access was no longer required, which may have contributed to the overall low CRBSI incidence. Dwell times > 10 days were associated with a higher risk of catheter infection for non-subclavian catheters in a large post-hoc analysis [10] and another study by Schalk et al. [11].

Interestingly, we observed a significantly lower rate of exit site infections in the PICC group compared to the CICC group (7.4 vs. 23.1 per 1000 catheter days), suggesting a potential advantage of peripheral insertion regarding local site complications. This observation is consistent with earlier studies indicating higher skin and catheter colonization rates in the cervical and thoracic area [12, 13] possibly resulting in higher exit site infections in CICCs. Given the prolonged neutropenia associated with AML induction therapy, reducing exit site infection risk may have important clinical implications. Interestingly, the higher rate of reported exit site infections did not result in higher rates of CRBSIs in the CICC group, probably because the CICCs were immediately removed in most cases if signs of exit site infections occurred.

Although bleeding events were more frequent in the PICC group (13.9 vs. 6.4 per 1000 catheter days), this difference was not statistically significant. All but one bleeding events were minor and not associated with adverse outcomes. It should also be noted that more patients in the CICC group received platelet transfusions at the time of catheter insertion, despite comparable platelet count in both groups. This most likely reflects a more cautious approach by clinicians due to the perceived higher bleeding risk associated with catheter placement in the cervical region. While platelet transfusions increase procedural safety, it also leads to higher consumption of platelet concentrates [14]. Consequently, catheter choice may have implications not only for patient safety but also for transfusion stewardship and healthcare resource utilization.

Other catheter-related complications, such as nerve injuries or catheter dysfunction, were rare in both groups. No pneumothorax occurred in either group. One case of nerve injury and two cases of symptomatic catheter-related venous thrombosis were observed in the PICC group. However, due to the retrospective design, asymptomatic or unreported thrombotic events may have gone undetected. This is particularly relevant given previous literature describing a higher incidence of catheter-associated thrombosis with PICCs compared to CICCs [7, 15]. As thrombosis screening in our cohort was performed only in symptomatic patients, the true incidence may have been underestimated. Consequently, the rather similar frequency in catheter-related thrombosis in both groups should be interpreted with caution, as the overall safety profile of PICCs with regard to thrombotic events may have been overestimated. All patients, however, consistently received thromboprophylaxis with low-molecular-weight heparin when platelet counts were > 30 × 10⁹/L, which may have reduced thrombotic risk.

Baseline differences between the groups likely reflect relevant clinical and logistical factors. Patients receiving CICCs presented with higher baseline inflammatory markers (CRP 3.7 vs. 1.8 mg/L), and in 21 patients (36.8%), CICC insertions occurred in the intermediate care unit (IMC) or intensive care unit (ICU). Importantly, nine of these were elective placements performed in these units because of better availability of experienced staff rather than acute clinical deterioration. In contrast, PICCs were exclusively inserted electively in the radiology department. These procedural and clinical differences likely influenced catheter choice and may have confounded outcome comparisons.

These differences in catheter placement settings also point out an important practical consideration: While PICCs, which typically have one or two lumens, are sufficient for daily clinical use in clinically stable AML patients, they may be inadequate in situations requiring rapid access for multiple infusions, such as during clinical deterioration. In such cases, the placement of an additional CICC may become necessary. This is particularly relevant as complications such as sepsis are common during AML induction therapy, and up to 20–30% of patients require at least one stay in the intensive care unit [16, 17].

This study has several limitations. First, it was conducted at a single center, and catheter selection was not randomized but based on physician judgment and clinical context, potentially introducing selection bias. Although baseline characteristics were largely comparable, residual confounding cannot be excluded. Factors that may have influenced catheter selection, such as vascular anatomy, vein quality, anticipated need for intensive care support, or additional patient-specific clinical considerations, were not systematically captured. Furthermore, the experience and individual preferences of the inserting physicians may also have influenced catheter choice beyond technical feasibility alone. Second, despite good documentation, the retrospective design limits data completeness and standardization of clinical practice. Third, the sample size was relatively small, limiting the power to detect differences in rare outcomes. Therefore, our findings should be considered hypothesis-generating and require confirmation in larger prospective studies.

In conclusion, PICCs were associated with significantly longer catheter dwell times and fewer exit-site infections while CRBSI rates were comparable to those of CICCs. These findings suggest that PICCs are a good alternative for clinically stable patients undergoing planned AML remission induction chemotherapy. Ultimately, the choice of central venous catheter should be individualized and primarily guided by the patient’s clinical condition and anticipated supportive care requirements.

## Data Availability

Due to data protection regulations and the conditions of the ethics approval for this retrospective study, the underlying patient-level data cannot be made publicly available. Anonymized data may be made available by the corresponding author (sabine.ehrlich@med.uni-muenchen.de) upon reasonable request, subject to institutional policies and ethics approval.
